# A Two-Step Approach to Orange Peel Waste Valorization: Consecutive Extraction of Pectin and Hesperidin

**DOI:** 10.3390/foods12203834

**Published:** 2023-10-19

**Authors:** Onofre Figueira, Verónica Pereira, Paula C. Castilho

**Affiliations:** CQM—Centro de Química da Madeira, Campus da Penteada, Universidade da Madeira, 9200-105 Funchal, Portugal; onofre.figueira@staff.uma.pt (O.F.); 2069318@student.uma.pt (V.P.)

**Keywords:** citrus waste, green chemistry, hesperidin, pectin

## Abstract

Citrus consumption translates into large amounts of residue, the disposal of which is associated with environmental issues and high costs. Current trends in citrus waste focus on the extraction of highly valued bioactive compounds via single-compound extraction. There is a lack of knowledge on how these methodologies can be introduced into extraction schemes of bioactive compounds, maximizing the residue potential and reducing its amount. The present work aimed to address this issue by designing a consecutive extraction of pectin and hesperidin from orange peel waste. A novel method for extraction and precipitation of hesperidin with an eco-friendly approach is also presented. After neutral pretreatment, pectin extraction was conducted under acidic conditions, followed by hesperidin extraction with a drastic pH change. Pectin had a high AUA content (66.20 ± 1.25%), meeting the criteria for use in the food industry. The best-tested conditions for hesperidin extraction (30 min, 70 °C, 1:10 (*w*/*v*)) provided a yield of 1% and a purity of 84%. The designed extraction scheme shows the potential of citrus waste as a source of bioactive compounds of good quality and high interest in the food industry while following the principles of green chemistry and circular economy.

## 1. Introduction

Vegetables and fruits are widely consumed foods worldwide as both direct and processed commodities. Large amounts of waste are produced as a result of this consumption, whether from household consumption or industrial processing [1,2]. Approximately 1.3 billion tons of food waste are produced globally per year, resulting in an estimated economic loss of 1 trillion dollars [3,4]. Citrus fruits are the most widely cultivated, consumed, and processed fruits in the world owing to their health benefits and organoleptic properties, being perceived as the most readily available source of vitamin C. They show antioxidative, anti-inflammatory, and neuroprotective properties [1]. Oranges are the most produced fruit, with 55% to 70% of the world’s total citrus consumption [2,5]. However, only one-third of cultivated citrus matter is processed in the citrus industry, producing around 50 to 60% of organic waste [6]. Peels, leftover pulp, and seeds are released into the environment, causing pollution problems, mostly because traditional disposal methods are insufficient, expensive, and harmful [7,8]. For example, their illegal discharge or incineration causes the release of methane and carbon oxide, contributing about 6% of the global greenhouse gases produced together with other wastes [9]. For example, these wastes act as an ideal substrate for developing potentially pathogenic organisms [10], including mycotoxin-producing fungi and insects [11]. Considering this reality, several studies have focused on recycling and reutilization, not only to reduce waste production and disposal costs but also to shine a light on the nutritional and economic value potential of these wastes [11]. These residues represent a source of several high-added-value compounds, such as dietary fiber (from which pectin is encompassed), minerals, organic acids, flavonoids (hesperidin, hesperetin, diosmin, narirutin), terpenes (limonene), and carotenoids, among others [6,12]. More recently, citric wastes have been used in the food and dietetic industries as an additive or as a prebiotic, but also for non-food applications, such as the production of biodegradable packaging materials and of pharmaceutical and cosmetic products with anti-aging properties [11,12].

Hesperidin (3′,5,7-trihydroxy-4′-methoxy-flavanone-7-O-rutinoside) is the most abundant glycosylated flavanone found in citrus fruits, especially in the albedos of sweet oranges and tangerines [8,13]. Over the past few decades, hesperidin has been extensively studied for its therapeutic applications and is currently considered an anti-inflammatory agent for, or assisting in, the reduction of leg swelling due to fluid accumulation [13]. This citrus flavanone has also been researched as a possible antiviral agent against the Influenza A virus [14], Hepatitis B virus [15], and SARS-CoV-2 [16]. Moreover, it has been studied as an anti-obesity [17], neuroprotective [18,19], anti-cancer [20], and anti-aging agent [21,22]. In the food industry, hesperidin derivatives are commonly used as food additives to enhance the antioxidant and antimicrobial activities of active packaging films instead of hesperidin, given its limited water solubility, bitterness, and poor oral bioavailability [23,24]. Even though hesperidin has been highly researched for its potential therapeutic and environmental applications, its extraction from different citrus matrices has proven to be a challenge. Therefore, the optimization of extraction methods is of equal importance to the therapeutic and environmental research. Hesperidin presents as a value-added product with a market value of about USD 101.3 million in 2021 and it is expected to reach USD 161.4 million by 2028 [25].

Pectin is a naturally occurring structural compound found in the primary cell walls and intracellular layers of plant cells, in particular in fruits such as citrus fruits. Pectin is found in various concentrations in fruit cells, depending on its function [26]. It is well-known for its role in plant growth as one of the primary cementing agents of cellulose fibrils, as well as its role in ion balance in plants and in the passage of nutrients and water [27,28]. In citrus, this fiber is primarily found in the peel (0.5 to 3.5 percent) and its synthesis involves multiple enzymes responsible for the formation of different glycosidic linkages and modifications in the glycosyl residues in the pectic chains [5,26]. Pectin is a linear polymer made up of hundreds to thousands of D-galacturonic acid monomeric units (around 70% of total pectin) linked by α-(1 → 4) glycosidic bonds and can be associated with different neutral sugars. Pectin, despite its common backbone, can have a variety of structures that change depending on the source and method of extraction. For example, the backbone can be replaced with rhamnopyranose units linked by α-(1 → 2) glycosidic bonds, resulting in galactose, mannose, glucose, and xylose side chains. Furthermore, galacturonic acid units may be methyl or acetyl esterified, resulting in two types of pectin: high methoxyl pectin and low methoxyl pectin [26,29], depending on having more than 50% or less than 50% esterified galacturonic acid moieties. The two groups show distinct properties and have different applications [5,26]. Pectins have been extensively researched due to their gelling properties, and they are frequently used as a gelling, thickening, and stabilizing agent in the food industry for the production of jams and jellies. In this industry, pectin has received increasing attention as a fully plant-based material substitute for gelatin and as a promising active food packaging film as it offers a good barrier against oxygen, high water vapor permeability, and antioxidant activity [30]. Because of its abundance, ease of obtaining, and biocompatibility, this polysaccharide has recently also been studied as a starting material to produce hydrogels. As a result, its application range has expanded to include the pharmaceutical and biomedical industries, hygiene, electronics, adhesives, paper substitutes, and environmental remediation fields [27,28,31]. Pectins are under a very active line of investigation as promising anti-cancer agents by acting as inhibitors of galactose binding by the protein galectin-3, thus reducing tumor cell aggregation and adhesion and stimulating apoptosis. Pectin also reduces angiogenesis, which helps reduce metastasis [32]. Pectin is one of the most abundant and valued hydrocolloids [33] with a market value of about USD 1.5 billion in 2022 and it is expected to reach UDS 2.2 billion by 2028 [34].

Different techniques are available for either hesperidin or pectin extraction from citrus or food waste. However, conventional extractions pose general environmental concerns. Pectin extractions are typically based on acidic hot extraction using acids with a high environmental impact, such as hydrochloric acid (HCl) or sulfuric acid, as well as long extraction times [27]. Recent studies have identified the same problem, searching for more green and eco-friendly methodologies for the obtention of citrus pectin [35]. Since hesperidin is insoluble in water, its extractions have traditionally been performed with organic solvents, especially methanol, which results in time-consuming repeated crystallizations and low purity. While effective, methanol is considered toxic and aggressive for the environment. In addition, most of the methods reported are non-selective for this flavonoid and, consequently, contribute to the presence of impurities that can make difficult hesperidin crystallization [36].

In the present work, we propose a method in line with the principles of green chemistry and circular economy and focus on the development and optimization of a green consecutive batch extraction of pectin and hesperidin, while simultaneously maximizing the reuse of orange waste and solvent. Hot acidic water extractions were performed for pectin, followed by recovery of the residues and assessment of the optimal temperature, time, and residue/solvent ratios for ethanol in alkaline conditions for hesperidin extractions. These working conditions also provided a new hesperidin precipitation method avoiding the use of other solvents. All ethanol and acetone involved in this study were recovered and reused in the following extractions.

## 2. Materials and Methods

### 2.1. Raw Material and Reagents

Orange peels were gathered from the university student’s bar in July 2022 and immediately treated. All reagents were of analytical grade. Deuterated methyl sulfoxide-d6 (DMSO-d6) was bought from Thermo Scientific (Reinach, Switzerland). HCl was bought from Honeywell (Offenbach, Germany), NaOH was bought from Fisher Scientific (Loughborough, UK), and acetone was bought from Fisher Scientific (Merelbeke, Belgium). Ethanol 96% was purchased from Aga (São Brás de Alportel, Portugal), which was only used in the first extraction, followed by recovery and distillation. Citric pectin was bought from Thermo Scientific (Reinach, Switzerland) and hesperidin standard was bought from Sigma-Aldrich (Steinheim, Germany) with 92.1 ± 1.9% purity. Both were used for comparative purposes.

### 2.2. Pretreatment and Washing

The fresh peels were washed and the flavedos and excess pulp were removed and discarded. After that, the albedos were cut into coarse pieces and submitted to two different treatments to remove low carbohydrates, color pigments, organic compounds, and inactive pectic enzymes [37]. Pretreatment was carried out by blanching in water at 90 °C for 1 h using a solid-to-liquid ratio of 1:25 (*w*/*v*). The mixture was then filtered using a food-grade 200 mesh nylon filter. Finally, the blanched albedos were dried in a food dehydrator at 50 °C until a constant weight was achieved, then mechanically ground using a domestic grinder before being packed in polyethylene bags and stored at room temperature. Before each extraction, the blanched albedos were washed with ethanol at 70 °C for 20 min using a solid-to-liquid ratio of 1:10 (*w*/*v*), resulting in the alcohol insoluble solids (AIS) used in the next steps [37]. The resulting ethanol was recovered, distilled, and reused for up to five further pretreatments and pectin and hesperidin extractions. 

### 2.3. Consecutive Extractions

(A) Pectin extraction

AISs were dried at 50 °C and an acidic extraction (pH around 1.5) was conducted using a 1:15 (*w*/*v*) solid-to-liquid ratio with an aqueous hydrochloric acid (HCl) solution, 0.1 N, at 75 °C for 1 h on a hotplate with constant stirring. The mixture was allowed to cool down to room temperature and then filtered. In total, 3 volumes of ethanol were added to the filtrate to promote pectin precipitation, resulting in a suspension that was left overnight at 8 °C. Pectin was recovered by centrifugation at 4000 rpm for 15 min, followed by filtration. Pectin was washed with distilled water twice with 2 volumes of acetone to remove pigments [38]. This acetone was distilled and reused in the following pectin-washing steps.

(B) Hesperidin extraction

**Figure 1:**
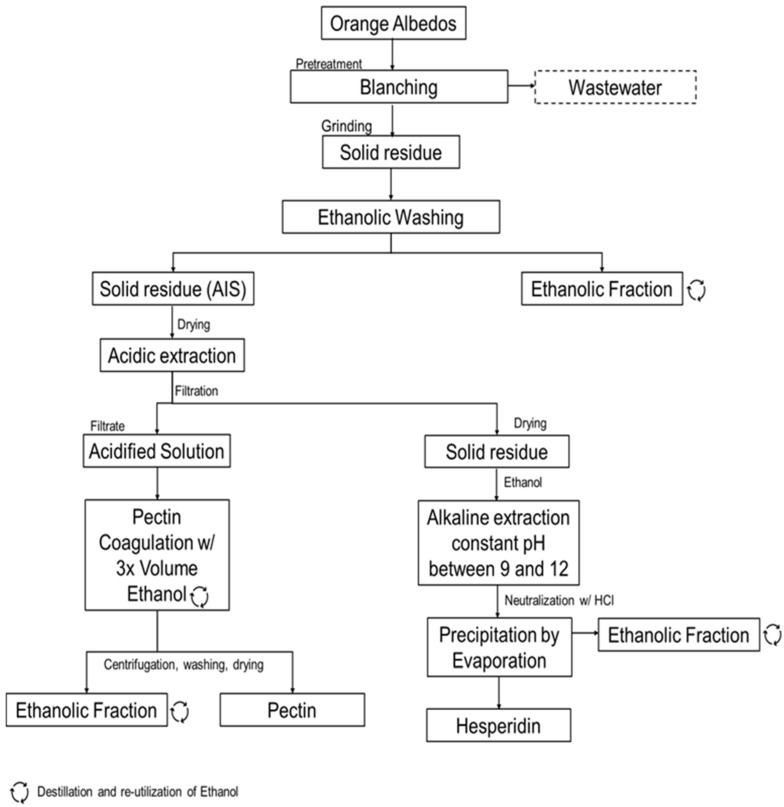
Consecutive extraction scheme of hesperidin and pectin from orange peel waste.

The solid fraction of the previous extraction was dried to a constant weight and was suspended in ethanol and basified to pH 9 to 12 to extract hesperidin [39]. The pH was maintained throughout the extraction by adding an aqueous solution of sodium hydroxide (NaOH) at 0.1 N and using a pH meter. This step was added since, in alkaline conditions, hesperidin phenol groups are deprotonated, which enhances hesperidin solubility in aqueous media [22]. This mixture was allowed to cool down to room temperature and was vacuum filtered using a Büchner funnel. The filtrate was neutralized with an aqueous HCl solution at 0.25 N until a pH of 6–7 was achieved to reduce hesperidin solubility by protonating its phenol groups. The ethanol was recovered in the rotatory evaporator, at 50 °C, which triggered the instant hesperidin precipitation. The suspension was kept overnight in the distillation flask at 8 °C to ensure that all hesperidin precipitated. Pale-yellow hesperidin was recovered using vacuum filtration with a sintered funnel and washed with cold distilled water and acetone. Finally, it was dried at 40 °C, until constant weight and stored in glass flasks at room temperature and in the dark. Figure 1 contains the representative diagram of the drawn consecutive extraction.

The extraction conditions evaluated in hesperidin extraction were temperature (50, 60, and 70 °C), the extraction time (30, 60, 120, and 180 min), and the solid-to-liquid ratio (1:10, 1:15, and 1:20 (*w*/*v*)). Each condition was tested in triplicate.

### 2.4. Statistical Analysis

The data obtained from the hesperidin yields were statistically analyzed by one-way analysis of variance (ANOVA) for each studied parameter (temperature, extraction time, and solid-to-liquid ratio), followed by Tukey’s multiple comparisons test (SPSS, Version 23.0, IBM Corporation, Armonk, NY, USA). Significance was accepted at *p* < 0.05.

### 2.5. Characterization

#### 2.5.1. Extractions Yields

The extraction yields were calculated by comparing the mass of the extracted compound to the initial mass of the whole albedo. This parameter measures the solvent’s extraction efficiency and the specific extraction from the starting material [40]. The extraction yield was calculated using the following formula.

(1) $$Compound\;Yield\;\left(\%\right)=\frac{Compound\;mass\;\left(g\right)}{Albedo\;dry\;powder\;\left(g\right)}\times 100$$

#### 2.5.2. Fourier Transform Infrared Spectroscopy

Pectin and hesperidin samples and respective commercial standards were characterized by Fourier Transform Infrared Spectroscopy (FTIR), using a PerkinElmer Spectrum Two spectrometer with the universal attenuated total reflection (UATR) attachment with pressure arm. All spectra were acquired by scanning in the range of 4000 and 400 cm^−1^ and performing 32 scans, with a resolution of 4 cm^−1^.

#### 2.5.3. Hesperidin Characterization

##### Nuclear Magnetic Resonance (NMR)

Extracted hesperidin and respective standards were analyzed by NMR. Approximately 20 mg were dissolved in 540 µL of deuterated dimethyl sulfoxide (DMSO-d6) and transferred to NMR tubes for further analysis.

NMR spectra were obtained with the Bruker Ultrashield 400 Plus equipment, running at a frequency of 400 MHz for ^1^H and 100 MHz for ^13^C. The time of acquisition of the spectra varied according to the type of analysis performed, being 50 s and 07:48 h for ^1^H and ^13^C, respectively. All spectra were acquired with 8 scans.

##### Melting Point

The melting point was determined for extracted and commercial hesperidin with the Melting Point M-560 equipment from Buchi. The measurement parameters were established as a temperature range of 190 to 270 °C, with a temperature gradient of 3 °C per minute.

##### HPLC-PDA

The purity of extracted hesperidin was determined using HPLC-PDA analysis. Both standard and extracted hesperidin solutions were prepared in triplicate at 100 µg/mL concentration, in a proportion of 1 mL of DMSO to 9 mL of ultrapure water. Solutions were filtered with 0.45 µm cellulose acetate filters and transferred to 1.5 mL vials. Separation of analytes was accomplished on a Kinetex 5u C18 LC column (150 × 4.60 mm) at a flow rate of 600 µL/min and injection volume of 20 µL. The gradient elution was composed of acetonitrile (mobile phase A) and distilled water with 0.1% acetic acid (mobile phase B). The mobile phase composition was 20% A for 3 min, increased to 30% at 3 min with a linear gradient, and then increased to 60% at 5 min with a second linear gradient. This composition was held for 5 min, followed by another increase to 90% A at 10 min and held for 2 min. The re-equilibration time was 8 min for each run. The monitoring UV wavelength was set at 280 nm.

##### 2.5.4. Pectin Characterization

###### Equivalent Weight

The pectin equivalent weight was calculated by adapting the work described by Siddiqui et al. (2021) [40]. As a result, 5 mL of ethanol was used to moisten 0.5 g of the pectin sample. In total, 1 g of NaCl was added to sharpen the endpoint, followed by 100 mL of distilled water with constant stirring until homogeneity was achieved. After adding 6 drops of phenol red, the solution was titrated against 0.1 N NaOH until turning bright pink when reaching the endpoint [41]. The titration was also controlled by a pH meter (endpoint around pH 7.5). The equivalent weight was calculated as follows:

(2) $$Equivalent\;Weight=\frac{Pectin\;mass\;\left(g\right)}{Volume\;NaOH\;\times Normality\;NaOH}$$

This parameter is then used to calculate the anhydrouronic acid content and the degree of esterification [42].

###### Methoxyl Content (MeO)

Titration was also used to determine the pectin’s methoxyl content. This was accomplished by adding 25 mL of 0.25 N NaOH to the previously neutralized solution obtained in the equivalent weight step. The mixture was thoroughly mixed and set aside for 30 min at room temperature. Following that, 25 mL of 0.25 N HCl was added, and the endpoint was titrated against 0.1 N NaOH [40,41]. The titration was once more also controlled by a pH meter. The methoxyl content was calculated using the equation below.

(3) $$Methoxyl\;content\;\left(\%\right)=\frac{Volume\;NaOH\;\times \;Normality\;of\;NaOH\;\times 31}{Pectin\;mass\;\left(g\right)\times 1000}\times 100$$

- 31 (g/mol) is the molecular weight of the methoxyl group.

###### Anhydrouronic Acid Content (AUA)

The anhydrouronic acid content was calculated using the equivalent weight and methoxyl content values, in the following equation, as reported by Maskey et al. (2018) based on Mohamed and Hasan (1995) [42,43].

(4) $$AUA\;\left(\%\right)=\frac{176\times 0.1z\times 100}{W\times 1000}+\frac{176\times 0.1y\times 100}{W\times 1000}$$

- 176 (g/mol) is the molecular weight of AUA.
- *z* is the volume of NaOH (mL) from equivalent weight determination.
- *y* is the volume of NaOH (mL) from methoxyl content determination.
- *W* is the mass of pectin (g).

The estimation of AUA is a critical parameter for determining purity, degree of esterification, and physical property evaluation [42].

###### Degree of Esterification (DE)

The degree of esterification was determined based on the MeO and AUA contents and calculated according to the following formula [42].

(5) $$DE\;\left(\%\right)=\frac{176\times MeO\%\times 100}{31\times AUA}$$

## 3. Results and Discussion

### 3.1. Development of Consecutive Extraction of Pectin and Hesperidin

The main goal of the present work was to develop a consecutive extraction process for two high-value bioactive compounds, the polysaccharide pectin and the flavonoid hesperidin, from the same batch of citrus waste. This was possible owing to differences in the solubility of these compounds since pectin extraction is favored in acidic conditions while hesperidin is extracted in alkaline ones. Pectin extraction must happen before hesperidin for a successful consecutive extraction because saponification and *β*-elimination occur at pH levels above six, making pectin isolation unfeasible [44]. Hesperidin extraction remains uncompromised due to its stability in acidic mediums [45].

**Figure 2:**
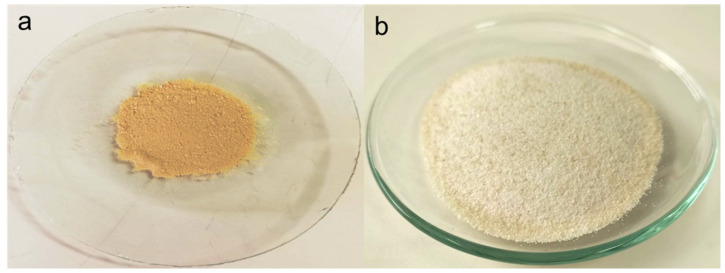
Obtained hesperidin (**a**) and pectin (**b**) employed the designed consecutive extraction.

The described methodology not only allowed for the isolation of pectin and hesperidin (Figure 2) but also for a more efficient precipitation and recovery of the latter. While evaporating ethanol, hesperidin initially dissolved in the hydroalcoholic solution precipitates owing to supersaturation. As a result, this hesperidin extraction methodology avoids long extraction and precipitation times, which are usually mentioned for alkaline extraction.

Orange peel waste is a byproduct generated in high quantities within the industrial sector. The implementation of a consecutive extraction process for pectin and hesperidin offers a promising strategy to optimize the utilization of this waste material, thereby mitigating waste generation, enhancing economic value, and expanding its applications in the food industry. Pectin and hesperidin, both obtained from orange peels, hold versatile functional properties, as previously mentioned. This approach not only promotes sustainability by reducing waste but also leverages the multifaceted benefits of pectin and hesperidin for improving product quality and shelf life.

### 3.2. Characterization

#### 3.2.1. Pectin Characterization

##### Extraction Yields

The yields of the obtained pectin varied from 17.23% to 20.61% (18.73 ± 1.19%). These yields are higher than the 8.78–15.79% [46] and 12.52–22.45% [47], both reported for pectin extracted from orange peels. The differences in the pectin yields may be related to orange maturation [48] or cultivar, as well as extraction methods applied.

##### Fourier Transform Infrared Spectroscopy

**Figure 3:**
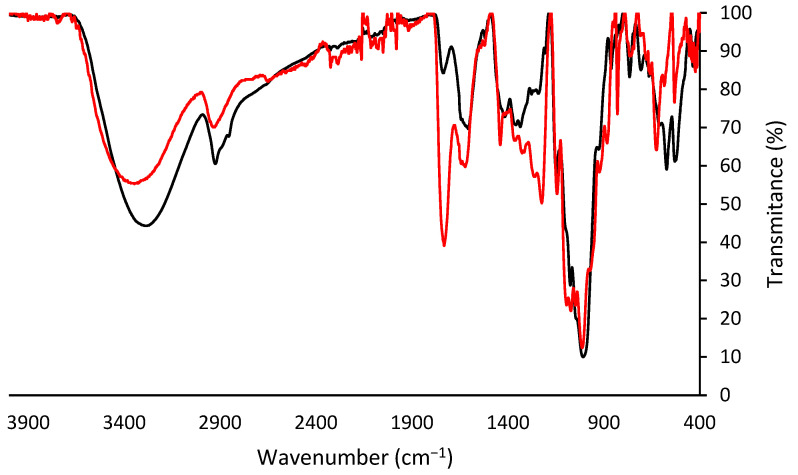
FTIR spectra analysis of commercial citric pectin and extracted citric pectin, represented in black and red, respectively.

The analysis of the functional groups of the obtained pectin (Figure 3) showed a peak between 3200 and 3500 cm^−1^ that is indicative of the presence of OH groups and a peak between 2800 and 3000 cm^−1^ attributed to C-H stretching vibrations, including CH, CH_2_, and CH_3_ stretching and bending patterns. Peaks in the areas of 1730 cm^−1^ and 1640 cm^−1^ were observed that are related to C=O stretching vibrations of esterified carboxyl groups and free carboxyl groups, respectively. Figure 3 shows the FTIR spectra of the extracted pectin and the commercial standard available at our lab, a pectin obtained from citrus. Another peak, at around 1300 cm^−1^ can be attributed to bending vibrations of the pyranose ring, and the peak in the area of 1200–1350 cm^−1^ can correspond to COO- stretching vibration of ester groups. Furthermore, characteristic pectin peaks around 1200–1000 cm^−1^ are attributed to the skeletal C-O and C-C vibrations due to the overlapped peaks of glycoside bonds and pyranose cycles. The peaks in the area of 830 and 500 cm^−1^ are associated with α- and β- configurations of the pectin. Overall, the FTIR spectra of the extracted pectin exhibited consistent functional groups with the standard, although with varying intensities in certain cases.

##### Equivalent Weight

The equivalent weight is dependent on the total content of free galacturonic acid in pectin, which is highly dependent on pH, extraction solvent, maturity state of the fruit, and the number of free acids present in pectin. This parameter also indicates gel-forming capacity; the greater the equivalent weight, the greater the gel-forming ability. High equivalent weight pectin has a higher gel-forming effect and low equivalent weight means higher partial degradation of pectin. In this study, the average equivalent weight obtained was 655.00 ± 21.41 g/mol, which was lower than the determined for the standard (5060 ± 11.78 g/mol). The obtained equivalent weight is in line with other published works, being higher than 381 g/mol for conventional extraction and 485 g/mol for microwave-assisted extraction for orange peels [46], and lower than 1744–1899 g/mol [47].

##### Methoxyl Content

The methoxyl content in pectin represents the mol amount of methyl alcohol in 100 mL of galacturonic acid. The MeO of this study’s extracted pectin was 6.92 ± 0.11 which is classified as low methoxy content pectin, since the content is less than 7%, but higher than the calculated for the standard (1.63 ± 0.42%). These values are in accordance with others reported for citrus, 5.75% and 6.99% for orange peels [46], between 6.7% and 7.1% for sweet lime [40], and are lower than the 9.06% reported for tangerine [48]. MeO of pectin represents the presence of free esterified carboxyl groups and can be affected by the extraction methods and the quality of the raw materials.

##### Anhydrouronic Acid Content

AUA content is related to the purity, quality, and gelling capacity of pectin to be applied for industrial purposes. The minimum value of 65% AUA is recommended by the FAO (Food and Agriculture Organization of the United Nations), FCC (Food Chemical Codex), and EU (European Union) for pectin to be used as commercial pectin [49]. The higher the AUA content, the higher the purity of the extracted pectin and the lower the amount of protein. In the present work, we obtained pectin with 66.20 ± 1.25% for the AUA, much higher than the estimate for the citric standard (12.75 ± 2.40%). This compares favorably to the recommendations of the FAO, FCC, and EU, as well as with the findings of other researchers reported. Nonetheless, this value was lower than the AUA of 82.0% reported for tangerine [48] and of 76.9%, 86.3%, and 100.3% for lemons, tangerine, and orange, respectively [50]. However, these late authors used peels from pristine fresh fruits.

##### Degree of Esterification

The degree of esterification is one of the most important properties for the application of pectin since it describes the ratio to which carboxyl groups in pectin coexist as methyl ester. In the industry, it is the parameter that indicates the gel-forming effect of pectin. When DE is greater than 50% pectin, it is categorized as high methoxyl pectin (HM), and when lower than 50%, it is categorized as low methoxyl content (LM). HM pectin tends to quickly form a gel at high temperatures and low pH, while LM pectin forms rigid gels by cross-linkage with calcium or multivalent cations [45]. The DE obtained in our study was found to be HM with 59.37 ± 0.75%, lower than the determined for the standard (71.68 ± 5.58%). These are in line with the other reported, being higher than 35.4–42.8% [46], slightly lower than 60.4% [49] and 73.2–77.5% [47]. The DE can differ depending on ripeness, origin, and extraction methods.

#### 3.2.2. Hesperidin Characterization

##### Extraction Yields 

The effect of different extraction times, temperatures, and ratios on hesperidin yields was evaluated (Figure 3). For each tested condition within a certain parameter, the other ones were maintained (70 °C and 1:10 (*w*/*v*), 30 min and 1:10 (*w*/*v*), and 30 min and 70 °C for the study of time, temperature, and ratio variation). 

Hesperidin yields showed no statistical difference (*p* > 0.05) between the studied extraction times. These results follow Flick’s second law, which predicts an equilibrium of solute concentration between matrix and solvent. Our findings are also supported by another study, which analyzed the effect of different extraction times (10, 20, 30, 40, 50, and 60 min) on hesperidin yields at 60% ethanol concentration, 60 °C, and 30 mL/g dry sample [51]. After 30 min of extraction, they verified that there was no statistically significant difference between yields. Given these observations, 30 min was chosen as the ideal extraction time.

**Figure 4:**
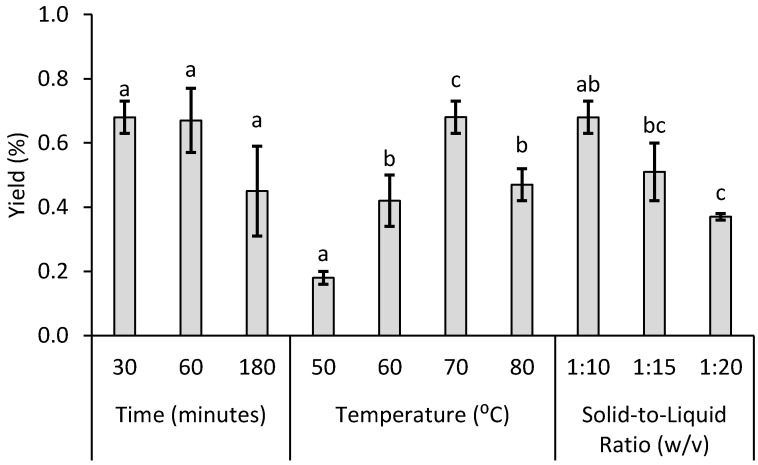
Effects of extraction parameters (time, temperature, and ratio) on hesperidin extraction yields. The statistical differences were analyzed using one-way ANOVA followed by Tukey’s multiple comparisons test. For each condition, each bar followed by the letters (a–c) is significantly different (*n* = 3; *p* < 0.05).

The temperature was the factor that affected the obtained yields the most (Figure 4). Hesperidin yields from the analyzed temperatures were statistically different (*p* < 0.05) between 50, 60, and 70 °C. At 80 °C, no statistical difference was seen when compared with 60 °C (*p* > 0.05). This result is explained by solvent evaporation during extraction owing to ethanol’s low boiling point (around 78 °C), resulting in hesperidin loss by precipitation. Other studies also studied the effect of different temperatures (25, 35, 45, 60, 75, 85, and 90 °C) [51]. They verified that hesperidin yields were significantly higher at 75 °C, followed by a decrease after 90 °C. As a result, 70 °C was chosen as the optimal temperature.

Solid-to-liquid ratio influence on hesperidin yields was the last evaluated parameter. The yield decreased with increasing the solid-to-liquid ratio with the only statistically significant difference being verified between the solid-to-liquid ratios 1:10 and 1:20 (*w*/*v*) (*p* < 0.05). This unexpected result could be understood by the fact that increasing quantities of NaOH were necessary to maintain a high alkaline pH for higher solid-to-liquid ratios. This could have been reflected in a higher deprotonation of other compounds (such as alcohol-soluble and lipophilic compounds) found in the same amount of solid. As a consequence, this impurity could have formed, for example, a flavonoid complex with hesperidin, which has already been reported as common in alkaline extractions, in higher amounts, thereby reducing its availability to crystallize [36]. Given these results, 1:10 (*w*/*v*) was fixed as the optimum ratio. In light of this situation, the best-tested conditions for hesperidin extraction were defined as 30 min, 70 °C, and 1:10 (*w*/*v*). 

##### Fourier Transform Infrared Spectroscopy

**Figure 5:**
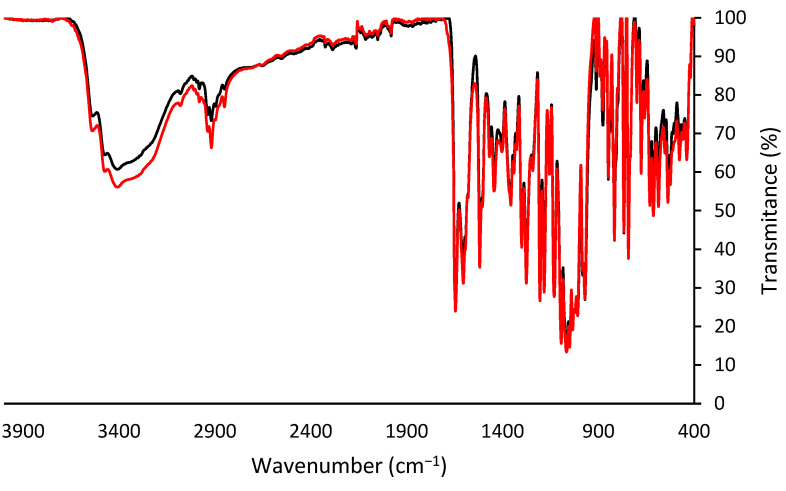
FTIR spectra analysis of hesperidin commercial standard and extracted, represented in black and red, respectively.

In the analysis of the FTIR spectrum of hesperidin, the hydroxyl stretching region shows a broad band at 3404 cm^−1^ (Figure 5), associated with intermolecular hydrogen bonds owing to the multiple hydroxyl groups present in the sugar moiety and also sharper bands at 3543 and 3473 cm^−1^. These bands represent the intramolecular hydrogen bonds between the hydroxyl and the ortho methoxy groups in ring B, and the hydroxyl group of ring A and the carbonylic oxygen of ring C. Furthermore, it observed all stretch bands referring to the CH bonds at 3083, 2983, 2938, and 2919 cm^−1^, and the hesperidin characteristic carbonylic bond located at 1645 cm^−1^. The bands at 1605, 1519, 1468, and 1443 cm^−1^ were attributed to the stretching of the C=C bonds from the phenolic rings, while the ones referring to the stretching of the C–O bonds were identified at 1275 and 1205 cm^−1^. The FTIR spectra of the extracted sample did not show the presence of impurities and the bands overlapped with the standard. These observations are in accordance with the FTIR analysis of hesperidin reported in other studies which were isolated with conventional methods such as hot methanolic maceration and Soxhlet extraction [8,13].

##### Nuclear Magnetic Resonance

**Figure 6:**
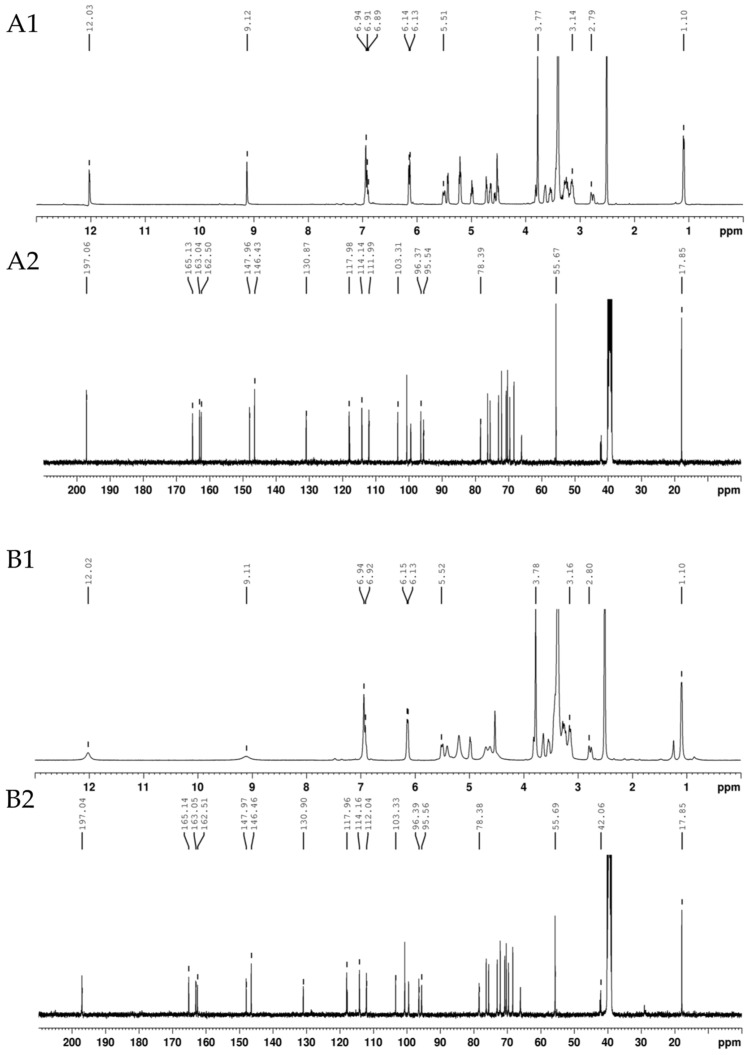
^1^H-NMR (1) and ^13^C-NMR (2) spectra of standard (**A1**,**A2**) and extracted hesperidin (**B1**,**B2**).

Owing to hesperidin solubility properties and solvent availability, NMR analysis was performed with DMSO-d6. This deuterated solvent shows a residual and an intense moisture peak at δ (ppm) 2.5 and 3.3, respectively. As can be seen in Figure 6(B1), these peaks overlap with some of the proton peaks of hesperidin, making them challenging to identify. Considering this situation, theoretical chemical shifts of protons from the aglycone hesperetin were calculated and their identification in the spectrum was attempted. The linkage between the aglycone and glycoside was corroborated by the identification of the only methyl group of rutinose. The same reasoning was applied to the interpretation of the ^13^C-NMR spectrum. Moreover, the ^1^H-NMR and ^13^C-NMR spectra of isolated hesperidin were compared with the ones of the standard.

^1^H-NMR (DMSO-d6, 400 MHz): δ (ppm) 12.02 (br. s, 1H, OH-5), 9.11 (s, 1H, OH-3′), 6.94–6.92 (m, 3H, H-2′, H-5′ and H-6′), 6.15–6.13 (m, 2H, H-8 and H-6), 5.52 (dd, 1H, H-2), 3.78 (s, 3H, MeO-4′), 3.16 (m, 1H, possibly H-3a), 2.80 (dd, 1H, H-3b), 1.10 (d, 3H, H-6‴).

^13^C-NMR (DMSO-d6, 100 MHz): δ (ppm) 197.04 (C-4), 165.14 (C-7), 163.05 (C-5), 162.51 (C-9), 147.97 (C-4′), 146.46 (C-3′), 130.90 (C-1′), 117.96 (C-6′), 114.16 (C-2′), 112.04 (C-5′), 103.33 (C-10), 96.39 (C-6), 95.56 (C-8), 78.38 (C-2), 55.69 (MeO-4′), 42.06 (C-3), 17.85 (C-6‴).

By comparing the ^1^H-NMR spectra of isolated and commercial hesperidin (Figure 6), it was possible to identify a proton signal at 1.24, absent in the standard. In addition, loss of signal definition was verified between 4.5 and 7.0 ppm. These findings suggest that the sample may contain some impurities that may be other flavonoids since extractions with NaOH followed by neutralization can allow the formation of flavonoid complexes [36].

##### Melting Point

The melting point determination of isolated hesperidin showed that it was less pure when compared with the respective standard. Extracted hesperidin exhibited a melting point range of 242.6–246.2 °C, while the commercial standard presented a fusion point of 249.8 °C. Impurities contribute to a lower melting point since they disrupt the crystalline structure of the solid, weakening the bonds that hold the atoms together. Consequently, less energy is needed to promote the phase transition in the parts of the solid surrounded by impurities. As a result, these findings support the previously observed ^1^H-NMR.

##### HPLC-PDA

**Figure 7:**
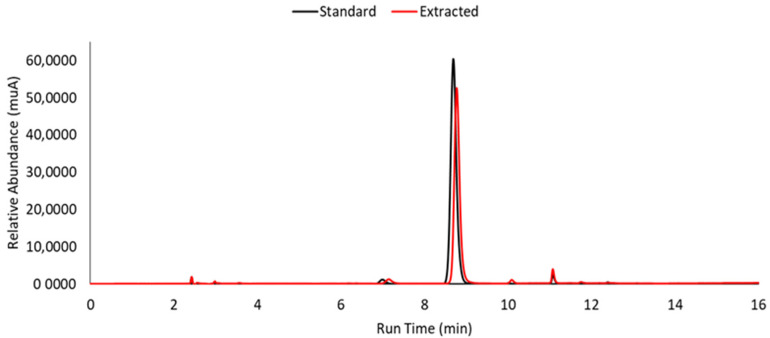
HPLC-PDA chromatogram analysis of hesperidin commercial standard and extracted, represented in black and red, respectively.

HPLC-PDA analysis of standard and extracted hesperidin showed the same major peak at 8.7 min retention time, as shown in Figure 7. Hesperidin purity was calculated by the ratio of the peak area relative to hesperidin and the total peak area. The obtained hesperidin showed a purity of 84.01%, which is close to the results of [8] which reported 89.4% for a methanolic extraction and to the standard, for which an 89% purity was determined (labeled as 92.1 ± 1.9%). Our methodology demonstrates that a similar purity can be obtained by using greener and safer solvents and is less time-consuming.

## 4. Conclusions

Orange peels are rich in bioactive compounds such as the flavanone glycoside hesperidin and the polysaccharide pectin. The development and optimization of a green approach to a consecutive extraction of pectin and hesperidin, with very satisfactory yields and purity and no further purification steps, while simultaneously maximizing the reuse of orange waste and solvents used was achieved in this work. The obtained HM pectin yields ranged between 17.23% and 20.61%, with a MeO, AUA, and DE of 6.92%, 66.20%, and 59.37%, respectively. Hesperidin extraction optimization was carried out over the pectin extraction residue with yields of 1% under optimized conditions. The hesperidin NMR, FTIR, and melting point revealed the presence of some minor impurities which can be attributed to other flavonoids. This issue can be addressed by recrystallization of the obtained hesperidin. Both ethanol and acetone used throughout the work were recovered and consecutively reintroduced in the consecutive optimization of the hesperidin extraction, following the principles of green chemistry and circular economy.

The development of this consecutive extraction took into consideration the extraction efficiency, the environmental impact, and the economic viability of this process. Citric acid, another value-added compound, could be obtained from the same residue and incorporated in this consecutive extraction to replace the hydrochloric acid used in pectin extraction. However, citric acid extraction and purification are dispendious, making its introduction economically unfeasible. Furthermore, the initial discarded flavedos and wastewater from the albedo’s blanching could be used for hydro distillation aiming at the extraction of essential oils from orange, rich in D-limonene. Despite being a methodology with high potential, the scale-up application of this extraction scheme needs to be studied to maintain the quality of the isolated compounds and that the amount of NaOH does not compromise the economic and environmental advantages of this process. The remaining solid residue could even be further exploited and applied for animal feeds. Contrary to other works, which focus on single compound extractions that also generate waste, our consecutive green pectin and hesperidin extractions maximize the potential of orange waste and prove to be a strong starting point for citrus wastes to be 100% converted into value-added products through efficient, economic, and eco-friendly batch methodologies.

## Data Availability

The data used to support the findings of this study can be made available by the corresponding author upon request.
